# The Cytotoxic Properties of* Baeckea frutescens* Branches Extracts in Eliminating Breast Cancer Cells

**DOI:** 10.1155/2019/9607590

**Published:** 2019-04-23

**Authors:** S. H. Shahruzaman, M. F. Mustafa, S. Ramli, S. Maniam, S. Fakurazi, S. Maniam

**Affiliations:** ^1^Department of Human Anatomy, Faculty of Medicine and Health Sciences, Universiti Putra Malaysia, 43400 UPM Serdang, Selangor Darul Ehsan, Malaysia; ^2^Faculty of Pharmacy, UiTM Cawangan Selangor, 42300 Puncak Alam, Selangor Darul Ehsan, Malaysia; ^3^Monash University, Wellington Road, Victoria 3800, Australia

## Abstract

Breast cancer is the leading cause of cancer death in women in over 100 countries worldwide and accounts for almost 1 in 4 cancer cases among women.* Baeckea frutescens *of the family Myrtaceae has been used in traditional medicine and is known to possess antibacterial, antipyretic, and cytoprotective properties. In this study, we investigated the role of* Baeckea frutescens* branches extracts against human breast cancer cells.* Baeckea frutescens* branches extracts were prepared using Soxhlet apparatus with solvents of different polarity. The selective cytotoxic activity and the glucose consumption rate of* Baeckea frutescens* branches extracts of various concentrations (20 to 160 ug/ml) at 24-, 48-, and 72-hour time points were studied using MTT and glucose uptake assay. The IC_50_ values in human breast cancer (MCF-7 and MDA-MB-231) and mammary breast (MCF10A) cell lines were determined. Apoptotic study using AO/PI double staining was performed using fluorescent microscopy. The glucose uptake was measured using 2-NBDG, a fluorescent glucose analogue. The phytochemical screening of major secondary metabolites in plants was performed. This study reports that* Baeckea frutescens* branches extracts showed potent selective cytotoxic activity against MCF-7 cells compared to MDA-MB-231 cells after 72 hours of treatment. Evidence of early apoptosis which includes membrane blebbing and chromatin condensation was observed after 72 hours of treatment with* Baeckea frutescens* branches extracts. Interestingly, for the glucose uptake assay, the inhibition was observed as early as 24 hours upon treatment. All* Baeckea frutescens* extracts showed the presence of major secondary metabolites such as tannin, triterpenoid, flavonoid, and phenol. However, alkaloid level was unable to be determined. The identification of* Baeckea frutescens* and its possible role in selectively inhibiting glucose consumption in breast cancer cells defines a new role of natural product that can be utilised as an effective agent that regulates metabolic reprogramming in breast cancer.

## 1. Introduction

*Baeckea frutescens* of the family Myrtaceae and subfamily Myrtoideae or locally referred to as* Cucur Atap *is a small tree which is found in Peninsular Malaysia and Sumatra [1] and distributed along the coastal areas of Southern China and Australia[2]. Traditional medicinal properties of* B. frutescens* were reported in treating influenza, dyspepsia, jaundice, dysentery, measles, and irregular menstrual cycles [3]. The bioactive constituents were shown to possess various properties such as antibacterial [4–6], antioxidant [7], and anti-inflammatory [8] ones as well as preventing arteriosclerosis by inhibiting LDL oxidation [9]. The cytotoxic effects of the methanolic derivatives were shown against leukaemic cells [10] and human lung, pancreatic, and breast cancer cells [6]. The hexane fraction* of B. frutescens *has been less extensively studied. Although little is known about the properties of the hexane fraction, it has recently been reported to possess moderate cytotoxic effect against human lung carcinoma [7].

To our knowledge, several metabolites were extracted from* B. frutescens *and structures of these metabolites were elucidated [1–28]. To date, there is yet no scientific research to elucidate the mechanism of* B. frutescens* in cancer cells. The aim of the study is to investigate the cytotoxic effect of* B. frutescens* branches extracts against breast cancer cells.

## 2. Materials and Methods

### 2.1. Extracts Preparation

*B. frutescens *or Cucur Atap was collected from Forest Research Institute Malaysia (FRIM) Research Station in Setiu, Terengganu, and its voucher specimen (Voucher number: KLU 47909) was deposited at Institute of Bioscience, Universiti Putra Malaysia. The branches of* B. frutescens* were cleaned and air-dried under shade for 7 consecutive days. The dried branches of* B. frutescens* were pulverized into coarse powder separately [7]. The powder was grounded and filtered using 0.9 mm filter membrane by vacuum pump to remove the debris and fibres. The coarse powder was extracted for 50% ethanol (B50), 70% ethanol (B70), 90% ethanol (B90), and water (WB) for 8 days, and finally the extractions were dried under a vacuum rotary evaporator (CCA-1110, Eyela, Tokyo, Japan).

### 2.2. Cell Lines

Human MCF-7 breast carcinoma cells (ATCC, USA) were grown as a monolayer in DMEM supplemented with 100 ug/ml streptomycin, 100 ug/ml penicillin, and 10% FBS. Human mammary breast cell lines, MCF10A cells (ATCC, USA), were cultured in DMEM/Ham's F-12 supplemented with 20 ng/ml epidermal growth factor (EGF), 0.01 mg/ml insulin, 500 ng/ml hydrocortisone, and 5% horse serum. Both cells were maintained at 37 °C in 5% CO_2_.

### 2.3. Determination of Cytotoxicity

The cytotoxicity of MCF-7 cells treated with* B. frutescens *extracts was determined by measuring the IC_50_ using the colorimetric MTT assay as previously described by Mosmann [29]. Briefly, cells were plated into 96-well microplates at a density of 5 × 10^3^ cells/well. Cells were cultured at 37 °C and treated at various time points (24-72 h) with various concentrations of extracts (0-1000 *μ*g/ml), and etoposide was used as a standard drug whilst DMSO was used as the vehicle control (control). The formazan grains were dissolved in DMSO, and the absorbance at 570 nm (Wavelength range: 550–600 nm) was read using an ELISA plate reader with the reference wavelength higher than 650 nm.

### 2.4. Glucose Consumption Assay

2-NBDG was used to measure glucose uptake as previously described by Hassanein et al. [30] Briefly, MCF-7 cells were seeded (0–30,000 cells/well) in clear-bottomed 96-well microplates in triplicates. Cells were allowed to adhere overnight at 37 °C before performing uptake assay. After overnight incubation, the cells were treated at three different concentrations; lower than IC_50_ value (low), IC_50_ value (IC50), and higher than IC_50_ value (high) extracts for 24, 48, and 72 h and DMSO was used as the vehicle control (control). The wells were washed twice with phosphate-buffered saline (PBS) and incubated with 2-NBDG (100 *μ*M) for 10 min at 37 °C in a humidified atmosphere of 5% CO_2_. The reaction was stopped by adding twofold volume of ice-cold PBS and the wells were rinsed with ice-cold PBS for three times. The fluorescent signals before (autofluorescence) and after adding 100 *μ*M 2-NBDG were measured (using the 485 nm_ex_ and 520 nm_emiss_ filter set). 2-NBDG uptake was also inhibited pharmacologically using WZB117 (10 *μ*M), a glucose transporter inhibitor (Sigma).

### 2.5. Acridine Orange and Propidium Iodide Staining

Cell death in MCF-7 cells was detected using propidium iodide (PI) (Sigma Aldrich, USA) and acridine-orange (AO) (Sigma Aldrich, USA) double staining and examined under fluorescence microscope. Briefly, treatment was performed in a 6-well plate. MCF-7 cells were plated at a concentration of 1×10^6^ cells/ml. The IC_50_ of the three extracts were used to treat the cells and DMSO was used as the vehicle control (control). Cells were then incubated in 37 °C in 5% CO_2_ for 24, 48, and 72 h. The cells were trypsinized and collected into centrifuge tubes and centrifuged at 1,000 ×* g* for 10 min. Supernatant was discarded and the cells were washed twice using PBS following centrifugation at 1,000 ×* g* for 10 min to remove the remaining media. In total, 10 *μ*l fluorescent dyes, AO (10 *μ*g/ml) and PI (10 *μ*g/ml), were added into the cellular pellet at equal volumes. Freshly stained cell suspension was dropped onto a glass slide and covered with coverslip. Slides were observed under ultraviolet- (UV-) fluorescence microscope (Olympus, Japan) within 30 min prior to the fading of the fluorescence colour.

### 2.6. Phytochemical Screening

10 *μ*L of* B. frutescens* branches extracts (1 mg/mL ethanol) was tested for the presence of tannins, alkaloids, triterpenoids, flavonoids, phenols, and alkaloids. The qualitative results were graded as very high (++++), high (+++), moderate (++), low (+), or not detected (-) based on the intensity of the coloured reaction observed compared with the positive control for the respective chemical reactions. The quantitative results for total content were further determined according to method done by Hisam et al. with slight modification [31]. The absorbance which corresponds to each test (Wavelength range: 435– 635 nm) was measured by using an ELISA plate reader (Tecan 200, Switzerland).

#### 2.6.1. Total Tannins Content

Five drops of 5% ferric chloride (Sigma Aldrich) were added to the extract. Formation of blue colour indicates the presence of tannins. 0.01 g/ml tannic acid (Sigma Aldrich) was used as the positive control. Absorbance was measured at 560 nm.

#### 2.6.2. Total Triterpenoids Content

The extract was mixed with chloroform and concentrated sulfuric acid was added to the solution. Formation of reddish brown colour at the interface indicates the presence of triterpenoids. 0.01 g/ml cholesterol (Sigma Aldrich) was used as the positive control. Absorbance was measured at 635 nm.

#### 2.6.3. Total Flavonoids Content

Drops of 10% lead acetate were added until formation of yellow precipitate. 0.01 mg/ml quercetin (Sigma Aldrich) was used as the positive control. Absorbance was measured at 590 nm.

#### 2.6.4. Total Phenols Content

The extract was mixed with 10% ferric chloride (Sigma Aldrich) solution and formation of bluish black color indicated the presence of phenols. 0.01 g/ml quercetin was used as the positive control. Absorbance was measured at 490 nm.

#### 2.6.5. Total Alkaloids Content

Drops of Dragendorff's reagent (Sigma Aldrich) were added to the extract. Formation of orange/orange reddish brown precipitate showed a positive result. 0.01 g/ml quinine sulfate (Sigma Aldrich) was used as the positive control. Absorbance was measured at 435 nm.

### 2.7. Statistical Analysis

Data were expressed as mean ± SEM (Standard Error of the Mean). A one-way analysis of variance (ANOVA), followed by Bonferroni post hoc test was employed for statistical analysis. SPSS (version 20) statistical software was used for the analysis of data and p < 0.01 was taken as the level of significance.

## 3. Results and Discussion

Two human breast carcinoma cell lines, MCF-7, which are oestrogen receptor positive (ER+) cells, and MDA-MB-231, which are oestrogen receptor negative (ER) cells, were used to determine the cytotoxicity of the* B. frutescens *branches extracts. Etoposide was used as the positive control. In this study, four extracts were prepared using ethanol (B50, B70, and B90) and water (WB) from the branches of* B. frutescens* using Soxhlet extraction method.

**Figure 1:**
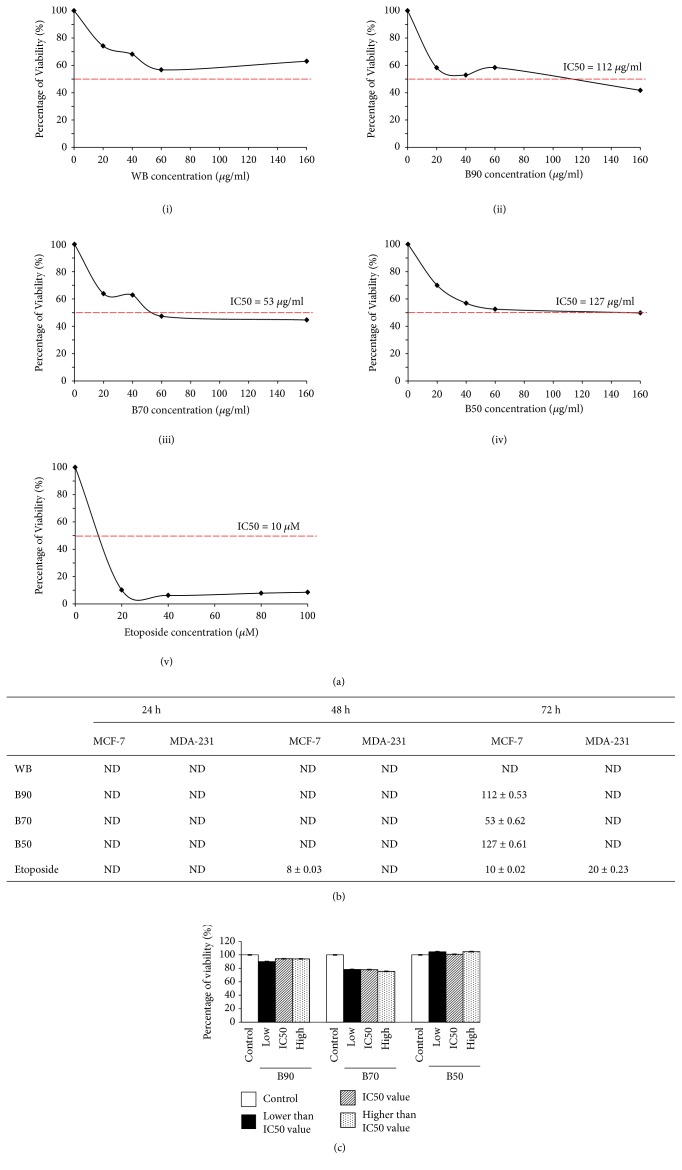
*Effects of B. frutescens branches extracts on MCF-7 cell viability*. (a) Cytotoxicity was determined using cell viability assay and the IC_50_ value was calculated as half maximal percentage of cell viability inhibition and it is indicated by the red horizontal line. MCF-7 cells were treated with either (i) WB, (ii) B90, (iii) B70, or (iv) B50 or (v) etoposide, which served as positive control for 72 h. Data is expressed as mean ± standard error mean based on six independent experiments with triplicate wells for each concentration. (b) IC_50_ values of B90, B70, B50, and WB against MCF-7 and MDA-MB 231 cells after 24, 48, and 72 h of incubation. (c) Normal mammary epithelial cells (MCF-10A) were treated with either B90, B70, or B50 at three different concentrations: lower than IC_50_ value (low), IC_50_ value (IC50), and higher than IC_50_ value (high) for 72 h. Data is expressed as mean ± standard error mean based on three independent experiments with triplicate wells for each concentration.

The viability of the human breast-derived cells after treatment with the* B. frutescens* branches extracts at 24, 48, and 72 h was estimated using MTT assay. No IC_50 _values were obtained from both cell lines at 24 h and 48 h of treatment. The cytotoxic effects of* B. frutescens* branches extracts were only seen at 72 h against MCF-7 cells. Among the four branches extracts tested, the water extract (WB) did not show cytotoxic activity against MCF-7 at any time point (Figure 1(a)). Amongst the ethanolic extracts, the most potent extract against MCF-7 cells was B70 with IC_50_ of 53 *μ*g/ml (Figure 1(b)). Data in this study indicates that the oestrogen receptor positive, MCF-7 breast cancer cells responded more favourably to* B.frutescens* than the triple negative MDA-MB-231 cells. Hence, MCF-7 cells were used for further cell death investigation (Figure 1(b)).

MCF10A, a normal human mammary epithelial cell line, was used as normal breast cell model and served as control. The* in vitro *cytotoxicity of* B. frutescens* extracts was evaluated against MCF10A at three various concentrations: a concentration lower than IC_50_, the IC_50_ value, and a concentration higher than IC_50_. All* B. frutescens *extracts tested showed insignificant cytotoxic activity against the normal human breast cancer cells (Figure 1(c)). This indicates the selective cytotoxic activity of* B. frutescens *towards cancer cells and not normal cells.

*B. frutescens* is traditionally known to have cytotoxic, anticariogenic, and antibacterial properties [4, 6, 10, 20]. Previous study found that baeckenones F, which was obtained from* B. frutescens* leaves, showed moderate cytotoxicity against MDA-MB-231 breast cancer cells [13, 22]. The cytotoxic activities of* B. frutescens* leaves were also reported in human leukemic cells [1, 10, 20] and human pancreatic ones [13, 22]. Many studies on* B. frutescens *were carried using the leaves and roots [8]. To date, this study is the first to examine the* in vitro* cytotoxic activity in the MCF-7 breast cancer cells treated with the* B. frutescens *branches extracts.

**Figure 2:**
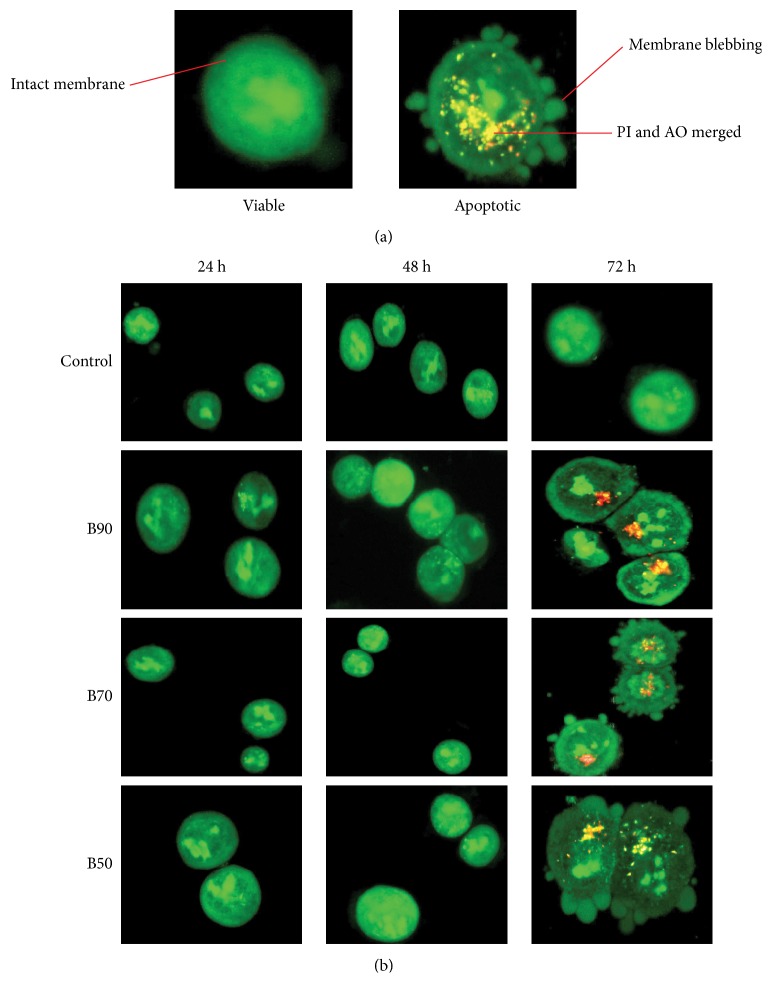
*Morphological observation of B. frutescens leaves extracts treated MCF-7 cells using AO/PI dual staining at X400 magnifications*. (a) Observation of morphological changes in MCF-7 cells. Viable cells are green stained cells with intact nucleus, condensed chromatin marked by intense green stained chromatin, membrane blebbing indicated by the outgrowth of plasma membrane and apoptotic cells are characterised by nuclear disintegration and leakage of plasma membrane. (b) MCF-7 cells were either treated without (untreated control) or with* B. frutescens* branches extracts (B90, B70, and B50) at their respective IC_50_ values and incubated for 24, 48, and 72 h (n= 3 independent experiments, magnification X100).

AOPI staining was used to determine the mode of cell death induced in* B. frutescens*-treated MCF-7 cells. AO is a nucleic acid selective fluorescent cationic dye that develops a protonated positive charge when it crosses the plasma membrane of viable and early apoptotic cells and intercalates into DNA and RNA to produce green fluorescence [32, 33]. Viable cells were stained green with intact nucleus structure (Figure 2(a)). Dead cells were stained red by PI, which penetrated the nuclear matter where the integrity of the cell membrane was compromised. All three ethanolic* B. frutescens *branches extracts did not show any morphological changes after 24 and 48 h treatment compared to control. Upon 72 h treatment, cells treated with IC_50_ value of* B. frutescens* branches extract exhibited membrane blebbing, chromatin condensation which indicates characteristics of early apoptosis (Figure 2(b)). Interestingly, the morphological changes observed corroborated with the data obtained from the MTT assay at which the IC_50_ was determined.

Most cancer cells exhibit an exceptionally high glycolysis rate and convert most incoming glucose to lactate in the presence of oxygen and this feature is known as Warburg effect. Warburg effect confers growth advantage to cancer cells when glucose supply is sufficient and this feature could be reflected as a fatal weakness of cancer cells when glucose supply is a problem [34]. Maximizing cancer cell glycolysis rate would possibly exhaust intratumoral glucose, leading cancer cell to death. It has been hypothesized that targeting glucose metabolism may provide a selective mechanism by which cancer cells are eliminated.

**Figure 3:**
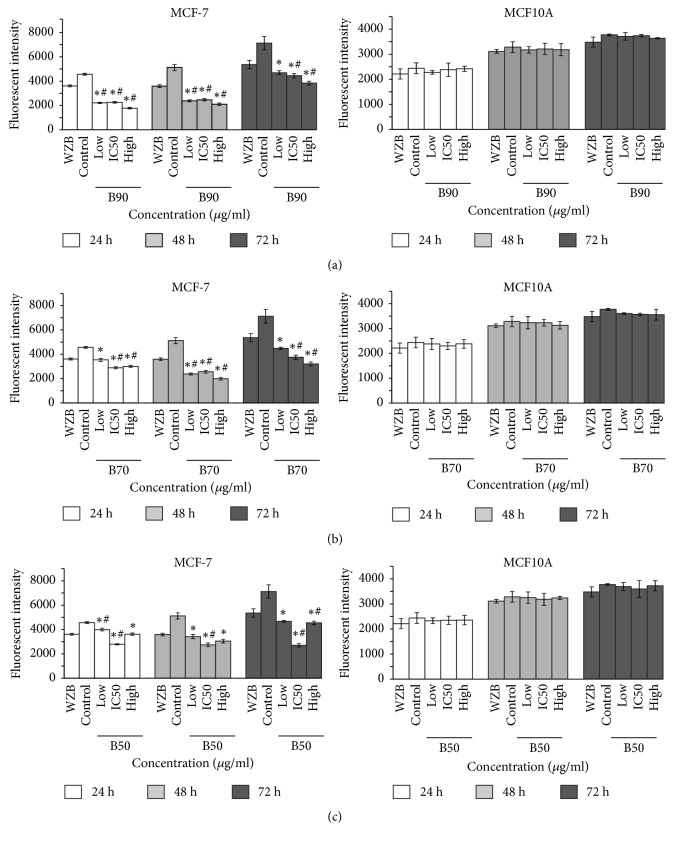
*Inhibition of glucose uptake of B. frutescens leaves extracts after 24, 48, and 72 h of incubation*. MCF-7 (left panels) or MCF10A (right panels) cells were treated with either (a) B90, (b) B70, or (c) B50 extract at three different concentrations: lower than IC_50_ value (low), IC_50_ value (IC50), and higher than IC_50_ value (high) for 24, 48, and 72 h. WZB117 served as positive control whilst DMSO is the vehicle control (control). Data is expressed as mean ± standard error mean based on four independent experiments with triplicate wells for each concentration. *∗*p< 0.01, compared with the untreated control (control); # p< 0.01, compared with the positive control (WZB117).

Glucose uptake was determined in MCF-7 cells treated with three* B. frutescens *branches extracts (B90, B70, and B50) at three different concentrations (lower than IC_50_ value, IC_50_ value, and higher than IC_50_ value) for 24, 48, and 72 h. WZB 117, a glucose transporter inhibitor that blocks the uptake of 2-NBDG via glucose transporter 1 (GLUT1), was used as positive control. After 24 h treatment, all branches extracts at their respective IC_50_ values significantly inhibited glucose uptake compared to WZB 117 and control. At 48 h of treatment, all three doses of B90 showed significant inhibition of glucose uptake compared to WZB 117 and control. After 72 h, B90 and B70 showed significant inhibition of glucose uptake compared to WZB 117. In summary, all* B. frutescens *branches extracts at all time points significantly inhibited glucose uptake compared to control (Figure 3). The extracts were also tested against MCF10A, human mammary breast cells. The results clearly showed that there were no significant glucose inhibitions in normal cells by all the extracts tested (Figure 3). This demonstrates that* B. frutescens* extracts selectively inhibit glucose uptake in MCF-7 cells.

The AO/PI staining and MTT assay showed that the IC_50_ concentrations of* B. frutescens *branches extracts induced cell death only after 72 h treatment. However, this did not correlate with glucose uptake inhibition which was observed as early as 24 h treatment. These results suggest that modulating glucose uptake is not the main mechanism of* B. frutescens *in inducing cell death. In the current study,* B. frutescens* extracts were found to inhibit glucose uptake in MCF-7 cells which lack in caspase 3. It is postulated that decreased glucose uptake observed in* B. frutescens*-treated cells might affect the mitochondria which is involved in inducing cell death. Mitochondria related caspase independent cell death can be triggered by various stimuli such as changes in the amount of ATP generated in the mitochondria and disruption of mitochondrial morphology which results in the release of intermembrane space proteins. The mechanism of* B. frutescens *extracts in modulating glucose metabolism warrants further investigation.

The preliminary phytochemical screening of* B. frutescens* was performed for tannin, triterpenoid, flavonoid, alkaloid, and phenol. The colour intensity or precipitate was used as analytical response for these tests. Presence of tannin, triterpenoid, flavonoid, alkaloid, and phenol were found in all extracts. All four* B. frutescens *branches extracts showed the presence of tannins, triterpenoids, flavonoid, and phenol except alkaloids (Table 1(a)). The highest tannin and phenol content was measured in B50 and B90, respectively. WB extract has the lowest tannin and phenol content among all four* B. frutescens* branches extracts. The triterpenoid and flavonoid content were the highest in B90 whilst these secondary metabolites were the lowest in WB extract (Table 1(a)). Ratio of quantitative estimation of tannin, triterpenoid, flavonoid, and phenol in* B. frutescens *is summarized in Table 1(b). Alkaloid was not included due to its low level compared to other secondary metabolites measured. Similar ratio profile of quantitative estimation of secondary metabolite was observed among the* B. frutescens* extracts tested. B70 and WB exhibited the same ratio profile of triterpenoids, flavonoids, and phenols content (1: 8: 3) (Table 1(b)). The variation activity among cell death and glucose uptake inhibition strongly suggests the presence of different types of secondary metabolites in the extracts

## 4. Conclusions

The cytotoxic activity of* B. frutescens* extracts against MCF-7 and MDA-MB-231 cells was determined at 72 h. However, the IC_50_ value was only obtained in MCF-7 cells as compared to MDA-MB-231 cells. Hence, the cytotoxicity of* B. frutescens* branches extracts was more potent in MCF-7 cells compared to MDA-MB-231 cells. The induction of cell death observed in MCF-7 cells was supported with the results obtained from AO/PI staining. Morphological changes showed evidence of early apoptosis in MCF-7 cells. All extracts selectively inhibited glucose uptake in breast cancer cells as early as 24 h after treatment. To our knowledge, this is the first study to report the role of* B. frutescens *branches extracts in modulating glucose in cancer cells. The preliminary secondary metabolites screening suggests that the branches extracts contain high flavonoids and undetectable level of alkaloids. However, the bioactive compound of* B. frutescens *branches extracts involved in eliminating breast cancer cells warrants further investigation.

## Figures and Tables

**Table 1a:** (a) Phytochemical constituent of *B. frutescens* branches in 1 *μ*g

Extracts	Tannin	Triterpenoids	Flavonoid	Alkaloids	Phenols
B90	168.41 ±0.05	162.30 ±0.17	481.17 ±0.16	ND	127.96 ±0.02
B70	150.52 ±0.04	46.37 ±0.04	367.00 ±0.02	ND	129.99 ±0.02
B50	206.19 ±0.02	152.30 ±0.19	254.50 ±0.12	ND	98.32 ±0.02
WB	90.41 ±0.03	13.41 ±0.06	104.50 ±0.03	ND	44.15 ±0.02

**Table 1b:** (b) Phytochemical ratio of *B. frutescens* branches in 1 *μ*g

Compound	Tannin	Triterpenoids	Flavonoid	Phenols
B90	1	1	4	1
B70	3	1	8	3
B50	2	2	3	1
WB	7	1	8	3

## Data Availability

The data used to support the findings of this study are available from the corresponding author upon request.

## Abbreviations

2-NBDG: 2-[N-(7-nitrobenz-2-oxa-1,3-diazol-4-yl) amino]-2-deoxy-d-glucose
AO: Acridine-orange
B50: * B. frutescens* branches prepared at the ratio of 1:50 in ethanol
B70: * B. frutescens* branches prepared at the ratio of 1:70 in ethanol
B90: * B. frutescens* branches prepared at the ratio of 1:90 in ethanol
DMEM: Dulbecco's Modified Eagle Medium
DMSO: Dimethyl sulfoxide
EGF: Epidermal growth factor
ER: Oestrogen receptor
GLUT1: Glucose transporter 1
IC_50_: Half maximal inhibitory concentration
MTT: 3-(4,5-Dimethylthiazol-2-yl)-2,5-diphenyltetrazolium bromide
PBS: Phosphate-buffered saline
PI: Propidium iodide
WB: Water extract.
